# *catena*-Poly[[bis­(pyridine-κ*N*)nickel(II)]-μ-oxalato-κ^4^ *O* ^1^,*O* ^2^:*O* ^1′^,*O* ^2′^]

**DOI:** 10.1107/S1600536808021703

**Published:** 2008-07-19

**Authors:** Zhen-Yu Xuan, Yong Che, Yong-Chun Huang

**Affiliations:** a Department of Laboratory and Equipment Management, Yanbian University, Yanbian 133002, People’s Republic of China

## Abstract

The title compound, [Ni(C_2_O_4_)(C_5_H_5_N)_2_]_*n*_, was synthesized under hydro­(solvo)thermal conditions. The Ni^II^ atom, lying on a twofold rotation axis, has an octa­hedral coordination geometry involving two N atoms from two pyridine ligands and four O atoms from two oxalate ligands. The Ni atoms are connected by the tetra­dentate bridging oxalate ligands into a one-dimensional zigzag chain.

## Related literature

For related literature, see: Lu *et al.* (1999 ▶); Vaidhyanathan *et al.* (2002 ▶); Wang *et al.* (2007 ▶); Yao *et al.* (2007 ▶).
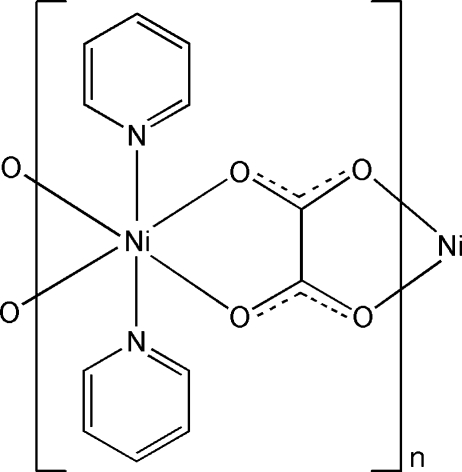


## Experimental

### 

#### Crystal data

[Ni(C_2_O_4_)(C_5_H_5_N)_2_]
*M* *_r_* = 304.93
Monoclinic, 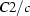

*a* = 14.357 (3) Å
*b* = 10.801 (2) Å
*c* = 8.6669 (17) Å
β = 91.52 (3)°
*V* = 1343.5 (5) Å^3^
*Z* = 4
Mo *K*α radiation
μ = 1.45 mm^−1^
*T* = 293 (2) K
0.26 × 0.24 × 0.22 mm

#### Data collection

Rigaku R-AXIS RAPID diffractometer
Absorption correction: multi-scan (*ABSCOR*; Higashi, 1995 ▶) *T*_min_ = 0.640, *T*_max_ = 0.726
6433 measured reflections
1519 independent reflections
1297 reflections with *I* > 2σ(*I*)
*R*_int_ = 0.041

#### Refinement

*R*[*F* ^2^ > 2σ(*F* ^2^)] = 0.042
*wR*(*F* ^2^) = 0.103
*S* = 1.04
1519 reflections
87 parameters
H-atom parameters constrained
Δρ_max_ = 0.73 e Å^−3^
Δρ_min_ = −0.30 e Å^−3^

### 

Data collection: *PROCESS-AUTO* (Rigaku, 1998 ▶); cell refinement: *PROCESS-AUTO*; data reduction: *PROCESS-AUTO*; program(s) used to solve structure: *SHELXS97* (Sheldrick, 2008 ▶); program(s) used to refine structure: *SHELXL97* (Sheldrick, 2008 ▶); molecular graphics: *SHELXTL* (Sheldrick, 2008 ▶); software used to prepare material for publication: *SHELXTL*.

## Supplementary Material

Crystal structure: contains datablocks global, I. DOI: 10.1107/S1600536808021703/hy2144sup1.cif

Structure factors: contains datablocks I. DOI: 10.1107/S1600536808021703/hy2144Isup2.hkl

Additional supplementary materials:  crystallographic information; 3D view; checkCIF report

## Figures and Tables

**Table:** 

Ni1—O2^i^	2.046 (2)
Ni1—O1	2.0716 (18)
Ni1—N1	2.081 (2)

**Table:** 

O2^i^—Ni1—O2^ii^	168.78 (10)
O2^i^—Ni1—O1	89.88 (7)
O2^ii^—Ni1—O1	81.96 (7)
O1—Ni1—O1^iii^	86.81 (11)
O2^i^—Ni1—N1	94.02 (8)
O2^ii^—Ni1—N1	93.66 (9)
O1—Ni1—N1	89.92 (9)
O1^iii^—Ni1—N1	174.81 (8)
N1—Ni1—N1^iii^	93.61 (13)

Symmetry codes: (i) 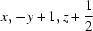; (ii) 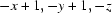; (iii) 
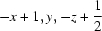
.

**Fig. 1.**
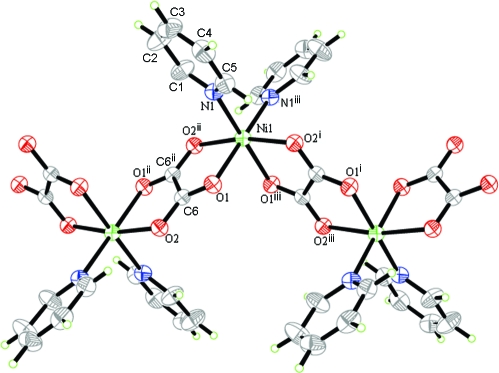
Part of the polymeric structure of the title compound. Displacement ellipsoids are drawn at the 30% probability level. [Symmetry codes: (i) x, 1-y, 1/2+z; (ii) 1-x, 1-y, -z; (iii) 1-x, y, 1/2-z.]

## supplementary crystallographic information

### Comment

Much research work has been done on metal–oxalate compounds, in the context of studies of molecular-based magnets and open framework structures (Lu *et al.*,  1999; Yao *et al.*,  2007). The geometrical coordination mode and strength of this ligand provide both rigidity and preferred coordination specificity for metal centers (Vaidhyanathan *et al.*,  2002; Wang *et al.*, 2007). In this paper, we report the hydro(solvo)thermal synthesis and structure of a new one-dimensional nickelous oxalate coordination polymer.

The title compound consists of one Ni^II^ atom lying on a twofold rotation axis, an oxalate ligand and two coordinated pyridine molecules (Fig. 1). The Ni^II^ atom exhibits a distorted octahedral geometry, defined by four O atoms of two oxalate ligands and two pyridine N atoms in a *cis* arrangement. The Ni—O distances are 2.046 (2) and 2.0716 (18) Å, while the O—Ni—O angles show distortions particularly as a result of chelation (Table 1). The tetradentate oxalate ligands link adjacent Ni atoms into a one-dimensional zigzag chain.

### Experimental

A mixture of K_2_C_2_O_4_.H_2_O (0.037 g, 0.2 mmol), H_3_BO_3_ (0.013 g, 0.2 mmol), NiCl_2_.2H_2_O (0.033 g, 0.2 mmol), KOH (0.012 g, 0.2 mmol), pyridine (4 ml) and water (8 ml) in a 25 ml Teflon-lined stainless steel reactor was heated from 298 to 393 K in 2 h and maintained at 393 K for 72 h. After the mixture was cooled to 298 K, blue crystals of the title compound were obtained.

### Refinement

H atoms were positioned geometrically and refined as riding atoms, with C—H = 0.93 Å and *U*_iso_(H) = 1.2*U*_eq_(C).

### Crystal data

**Table:**

[Ni(C_2_O_4_)(C_5_H_5_N)_2_]	*F*_000_ = 624
*M**_r_* = 304.93	*D*_x_ = 1.508 Mg m^−^^3^
Monoclinic, *C*2/*c*	Mo *K*α radiation λ = 0.71073 Å
Hall symbol: -C 2yc	Cell parameters from 1532 reflections
*a* = 14.357 (3) Å	θ = 3.3–27.5º
*b* = 10.801 (2) Å	µ = 1.45 mm^−^^1^
*c* = 8.6669 (17) Å	*T* = 293 (2) K
β = 91.52 (3)º	Block, blue
*V* = 1343.5 (5) Å^3^	0.26 × 0.24 × 0.22 mm
*Z* = 4	

### Data collection

**Table:**

Rigaku R-AXIS RAPID diffractometer	1519 independent reflections
Radiation source: rotating anode	1297 reflections with *I* > 2σ(*I*)
Monochromator: graphite	*R*_int_ = 0.041
Detector resolution: 10.0 pixels mm^-1^	θ_max_ = 27.5º
*T* = 293(2) K	θ_min_ = 3.3º
ω scans	*h* = −18→18
Absorption correction: multi-scan(ABSCOR; Higashi, 1995)	*k* = −13→13
*T*_min_ = 0.640, *T*_max_ = 0.726	*l* = −11→10
6433 measured reflections	

### Refinement

**Table:**

Refinement on *F*^2^	Secondary atom site location: difference Fourier map
Least-squares matrix: full	Hydrogen site location: inferred from neighbouring sites
*R*[*F*^2^ > 2σ(*F*^2^)] = 0.042	H-atom parameters constrained
*wR*(*F*^2^) = 0.103	*w* = 1/[σ^2^(*F*_o_^2^) + (0.0487*P*)^2^ + 1.5041*P*] where *P* = (*F*_o_^2^ + 2*F*_c_^2^)/3
*S* = 1.04	(Δ/σ)_max_ < 0.001
1519 reflections	Δρ_max_ = 0.73 e Å^−^^3^
87 parameters	Δρ_min_ = −0.30 e Å^−^^3^
Primary atom site location: structure-invariant direct methods	Extinction correction: none

### Fractional atomic coordinates and isotropic or equivalent isotropic displacement parameters (Å^2^)

**Table:**

	*x*	*y*	*z*	*U*_iso_*/*U*_eq_	
Ni1	0.5000	0.35667 (4)	0.2500	0.04167 (19)	
O1	0.57949 (11)	0.49601 (17)	0.1553 (2)	0.0468 (4)	
O2	0.57299 (12)	0.62481 (16)	−0.0453 (2)	0.0465 (4)	
N1	0.59041 (14)	0.2248 (2)	0.1633 (3)	0.0491 (5)	
C1	0.5679 (2)	0.1569 (3)	0.0391 (5)	0.0726 (10)	
H1	0.5088	0.1664	−0.0059	0.090*	
C2	0.6274 (3)	0.0743 (4)	−0.0251 (6)	0.0911 (13)	
H2	0.6085	0.0290	−0.1116	0.090*	
C3	0.7145 (3)	0.0584 (3)	0.0378 (5)	0.0790 (11)	
H3	0.7558	0.0019	−0.0040	0.090*	
C4	0.7395 (2)	0.1274 (4)	0.1630 (5)	0.0725 (10)	
H4	0.7986	0.1190	0.2083	0.090*	
C5	0.6764 (2)	0.2103 (3)	0.2231 (4)	0.0592 (8)	
H5	0.6946	0.2577	0.3083	0.090*	
C6	0.54390 (16)	0.5345 (2)	0.0324 (3)	0.0405 (5)	

### Atomic displacement parameters (Å^2^)

**Table:**

	*U*^11^	*U*^22^	*U*^33^	*U*^12^	*U*^13^	*U*^23^
Ni1	0.0290 (2)	0.0460 (3)	0.0499 (3)	0.000	−0.00167 (18)	0.000
O1	0.0341 (9)	0.0554 (11)	0.0503 (11)	−0.0087 (7)	−0.0082 (8)	0.0032 (8)
O2	0.0358 (9)	0.0508 (10)	0.0527 (11)	−0.0083 (7)	−0.0047 (8)	0.0002 (8)
N1	0.0351 (11)	0.0486 (12)	0.0637 (15)	0.0030 (9)	0.0034 (10)	0.0006 (10)
C1	0.0480 (17)	0.074 (2)	0.096 (3)	0.0063 (15)	−0.0007 (17)	−0.0296 (19)
C2	0.067 (2)	0.089 (3)	0.118 (3)	0.007 (2)	0.011 (2)	−0.043 (3)
C3	0.060 (2)	0.068 (2)	0.110 (3)	0.0157 (17)	0.025 (2)	−0.009 (2)
C4	0.0438 (16)	0.086 (2)	0.088 (3)	0.0185 (15)	0.0097 (16)	0.021 (2)
C5	0.0401 (14)	0.070 (2)	0.067 (2)	0.0114 (13)	0.0028 (13)	0.0074 (15)
C6	0.0293 (11)	0.0451 (13)	0.0470 (14)	−0.0015 (10)	0.0014 (10)	−0.0054 (11)

### Geometric parameters (Å, °)

**Table:**

Ni1—O2^i^	2.046 (2)	C1—C2	1.364 (5)
Ni1—O2^ii^	2.046 (2)	C1—H1	0.9300
Ni1—O1	2.0716 (18)	C2—C3	1.362 (6)
Ni1—O1^iii^	2.0716 (18)	C2—H2	0.9300
Ni1—N1	2.081 (2)	C3—C4	1.357 (6)
Ni1—N1^iii^	2.081 (2)	C3—H3	0.9300
O1—C6	1.240 (3)	C4—C5	1.385 (5)
O2—C6	1.263 (3)	C4—H4	0.9300
O2—Ni1^ii^	2.046 (2)	C5—H5	0.9300
N1—C1	1.335 (4)	C6—C6^ii^	1.556 (4)
N1—C5	1.336 (3)		
			
O2^i^—Ni1—O2^ii^	168.78 (10)	C5—N1—Ni1	121.3 (2)
O2^i^—Ni1—O1	89.88 (7)	N1—C1—C2	123.1 (3)
O2^ii^—Ni1—O1	81.96 (7)	N1—C1—H1	118.4
O2^i^—Ni1—O1^iii^	81.96 (7)	C2—C1—H1	118.4
O2^ii^—Ni1—O1^iii^	89.88 (7)	C3—C2—C1	119.9 (4)
O1—Ni1—O1^iii^	86.81 (11)	C3—C2—H2	120.1
O2^i^—Ni1—N1	94.02 (8)	C1—C2—H2	120.1
O2^ii^—Ni1—N1	93.66 (9)	C4—C3—C2	118.1 (3)
O1—Ni1—N1	89.92 (9)	C4—C3—H3	121.0
O1^iii^—Ni1—N1	174.81 (8)	C2—C3—H3	121.0
O2^i^—Ni1—N1^iii^	93.66 (9)	C3—C4—C5	119.7 (3)
O2^ii^—Ni1—N1^iii^	94.02 (8)	C3—C4—H4	120.2
O1—Ni1—N1^iii^	174.81 (8)	C5—C4—H4	120.2
O1^iii^—Ni1—N1^iii^	89.92 (9)	N1—C5—C4	122.4 (3)
N1—Ni1—N1^iii^	93.61 (13)	N1—C5—H5	118.8
C6—O1—Ni1	111.39 (15)	C4—C5—H5	118.8
C6—O2—Ni1^ii^	111.64 (15)	O1—C6—O2	125.6 (2)
C1—N1—C5	116.8 (3)	O1—C6—C6^ii^	117.5 (3)
C1—N1—Ni1	121.8 (2)	O2—C6—C6^ii^	116.9 (3)

Symmetry codes: (i) *x*, −*y*+1, *z*+1/2; (ii) −*x*+1, −*y*+1, −*z*; (iii) −*x*+1, *y*, −*z*+1/2.
